# Recent progress of the genetics of amyotrophic lateral sclerosis and challenges of gene therapy

**DOI:** 10.3389/fnins.2023.1170996

**Published:** 2023-05-12

**Authors:** Hui Wang, LiPing Guan, Min Deng

**Affiliations:** ^1^Institute of Medical Innovation and Research, Peking University Third Hospital, Beijing, China; ^2^Laboratory of Genomics and Molecular Biomedicine, Department of Biology, University of Copenhagen, Copenhagen, Denmark

**Keywords:** amyotrophic lateral sclerosis, genetics, gene therapy, motoneuron disease, neurogenetics, antisense oligonucleotide

## Abstract

Amyotrophic lateral sclerosis (ALS) is a neurodegenerative disorder characterized by the degeneration of motor neurons in the brain and spinal cord. The causes of ALS are not fully understood. About 10% of ALS cases were associated with genetic factors. Since the discovery of the first familial ALS pathogenic gene SOD1 in 1993 and with the technology advancement, now over 40 ALS genes have been found. Recent studies have identified ALS related genes including ANXA11, ARPP21, CAV1, C21ORF2, CCNF, DNAJC7, GLT8D1, KIF5A, NEK1, SPTLC1, TIA1, and WDR7. These genetic discoveries contribute to a better understanding of ALS and show the potential to aid the development of better ALS treatments. Besides, several genes appear to be associated with other neurological disorders, such as CCNF and ANXA11 linked to FTD. With the deepening understanding of the classic ALS genes, rapid progress has been made in gene therapies. In this review, we summarize the latest progress on classical ALS genes and clinical trials for these gene therapies, as well as recent findings on newly discovered ALS genes.

## 1. Introduction

Amyotrophic lateral sclerosis (ALS) is a rare, progressive, neurodegenerative disease, which is characterized by the degeneration of upper and lower motor neurons. It will lead to muscle weakness and paralysis (Brown and Al-Chalabi, 2017). The lifetime risk of ALS is approximately one per 350 people (Ryan et al., 2019; Feldman et al., 2022) and the incidence of ALS in European and American populations is two to three cases per year per 100,000 of the general population (Chio et al., 2013). Patients usually die within three to 5 years following the symptom onset. Currently, ALS is classified into familial and sporadic. Approximately 10–15% of ALS cases are considered “familial ALS” (FALS) and inherited in either an autosomal dominant, autosomal recessive, or X-linked mode, while the remaining are sporadic (SALS). About 70% of FALS and 15% of SALS have mutations in known ALS genes, including SOD1, FUS, TARDBP, C9ORF72, ATXN2, and so on (Table 1). Recently, 12 novel genes–ANXA11, ARPP21, CAV1, C21ORF2, CCNF, DNAJC7, GLT8D1, KIF5A, NEK1, SPTLC1, TIA1, and WDR7 have been identified using Genome-Wide Association Study (GWAS) and Whole Exome Sequencing (WES). These genes mainly affect protein homeostasis, DNA repair, RNA metabolism, vesicle transport, mitochondrial function, and other aspects. The core pathological change in ALS is motor neuron death in the motor cortex and spinal cord, the major neuropathological findings are intracellular cytoplasmic inclusions of eosinophilic Bunina bodies and ubiquitinated TDP-43 (Brown and Al-Chalabi, 2017). Pathological pathways for neuronal death include impaired RNA metabolism, altered proteostasis or autophagy, cytoskeletal or trafficking defects, mitochondrial dysfunction, and compromised DNA repair (Goutman et al., 2022a).

**Table 1:** Genes associated with amyotrophic lateral sclerosis.

Year	Gene	Chromosomal locus	Mode of inheritance	Overlay	Probable functions
2021	SPTLC1	9q22.31	AD	ALS; HSP; CMT	Sphingolipid synthesis
2020	WDR7	18q21	Unknow	ALS	Calcium flux; neurotransmitter release
2020	CAV1	7q31.2	Unknow	ALS	Intracellular calcium homeostasis
2019	GLT8D1	3p21.1	AD	ALS	Ganglioside synthesis
2019	ARPP21	3p22.3	Unknown	ALS	Toxic factor acts synergistically with GLT8D1 mutation
2019	DNAJC7	17q21.2	AD/AR	ALS	Protein homeostasis
2018	KIF5A	12q13.3	AD	ALS; HSP; CMT2	Intracellular traffic; axonal defect
2017	TIA1	2p13.3	AD	ALS	TDP-43 accumulation; RNA metabolism
2017	ANXA11	10q22.3	Unknown	ALS; FTD	Phospholipid and calcium-binding
2016	CCNF	16p13.3	AD	ALS; FTD	Autophagy; axonal defects; protein aggregation
2016	NEK1	4q33	AD	ALS	Cell cycles; DNA damage repair; cilia formation
2016	C21ORF2(also known as CFAP410)	21q22.3	AD	ALS	Cilia formation; DNA damage repair; mitochondrial function
2015	TBK1	12q14.2	AD	ALS; FTD	Autophagy; neuroinflammation
2014	CHCHD10	22q11.23	AD	ALS; FTD; ataxia	Mitochondrial function
2014	MATR3	5q31.2	AD	ALS; FTD	Ribostasis
2014	TUBA4A	2q35	AD	ALS; FTD	Cytoskeletal organization; axonal transport
2013	ERBB4	2q34	AD	ALS	Neuronal development
2013	HNRNPA2B1	7p15	AD; risk factor	ALS; myopathy; cognitive impairment	Ribostasis
2013	HNRNPA1	12q13:13	AD; risk factor	ALS; myopathy; cognitive impairment	Ribostasis
2012	ATXN1	6p22.3	risk factor	ALS	Nucleocytoplasmic transport
2012	EPHA4	2q36.1	Unknown	ALS; ataxia	Axonal degeneration
2012	PFN1	17p13	AD	ALS	Cytoskeletal organization; axonal grow and transport
2011	C9ORF72	9p21	AD	ALS; FTD	Intracellular trafficking; autophagy; protein stasis; nucleocytoplasmic transport
2011	SQSTM1	5q35	AD	ALS; FTD	Autophagy; neuroinflammation
2011	UBQLN2	Xp11	X-linked AD	ALS; FTD	Protein stasis
2010	SIGMAR1	9p13.3	AD/AR	ALS; FTD	Proteasome impairment; intracellular trafficking
2010	ATXN2	12q24	AD	ALS; SCA2	Ribostasis
2010	OPTN	10p13	AD/AR	ALS; FTD	Autophagy; neuroinflammation
2010	SPG11	15q14	AR	ALS; HSP; CMT	DNA damage
2010	VCP	9p13	AD	ALS; FTD	Protein stasis
2006	ANG	14q11	Risk factor	ALS; FTD	Angiogenesis
2009	Fig 4	6q21	AD	ALS	Intracellular trafficking
2009	UNC13A	19p13.11	Unknown	ALS; FTD	synapse function
2009	ELP3	8p21	Unknown	ALS	Ribostasis; cytoskeletal integrity
2009	FUS	16p11	AD/AR	ALS; FTD	Ribostasis
2008	TARDBP	1p36	AD/AR	ALS; FTD	Ribostasis
2006	CHMP2B	3p11	AD	ALS; FTD	Protein stasis; vesicular trafficking
2004	HFE	6p22.2	Unknown	ALS; Alzheimer’s disease; PD	Iron homeostasis
2004	VAPB	20q13	AD	ALS	Protein stasis
2003	DCTN1	2p13	AD; risk factor	ALS	Axonal transport
2001	ALS2	2q33	AR	ALS	Vesicular trafficking
1998	SETX	9q34	AD	ALS	Ribostasis
1994	NEFH	22q12	AD; risk factor	ALS	Axonal transport
1993	SOD1	21q22	AD/AR	ALS	Protein stasis; oxidative stress

AD, Autosomal dominant; AR, autosomal recessive; ALS, Amyotrophic Lateral Sclerosis; FTD, Frontotemporal Dementia; CMT2, Charcot–Marie-Tooth type 2 hereditary neuropathy; SCA2, Spinocerebellar Ataxia 2; HSP, Hereditary Spastic Paraplegia; PD, Parkinson’s disease.

The initial diagnostic criteria date back to El Escorial in 1990 (Brooks, 1994), later in 1998 El Escorian (Airlie House) was revised to improve sensitivity. Then in 2008 Awaji criteria were proposed, it suggests the use of electronic diagnostic research in the diagnosis to detect disease at an early stage (De Carvalho et al., 2008). They categorized the diagnosis of ALS as possible, probable, and definite based on the number of segments affected and combined with clinical or electrophysiological results. However, these criteria have some weaknesses, in particular lack of sensitivity, great complexity, and the use of diagnostic categories that are not related to the disease process. Therefore in 2020 The Gold Coast Standard has been proposed to simplify and possibly replace the revised El Escorial (Shefner et al., 2020). The simplified criteria abandon previous diagnostic categories and facilitate early and definitive diagnosis. Multiple studies have found that Gold Coast criteria are more sensitive and specific for identifying progressive muscular atrophy and excluding primary lateral sclerosis. Some researchers expect the new Gold Coast standard will help diagnose and remove uncertainty and confusion for patients and their families (Hannaford et al., 2021).

Because of the heterogeneity of clinical manifestations and clinical overlap with other more common neurological and neuromuscular diseases of ALS, its diagnosis is challenging. Now there is no special test to diagnose ALS, its clinical diagnosis is based on a history of progressive weakness, examination findings of both upper and lower motor neuron dysfunction, electromyography, laboratory testing, et al. (Oskarsson et al., 2018). With the deepening understanding of pathogenic mechanisms, new scoring systems, biological markers, electrophysiological tests, etc. have improved the accuracy of ALS diagnosis (Goutman et al., 2022b). Currently, doctors usually use the Amyotrophic Lateral Sclerosis Functional Rating Score Revised (ALSFRSR) to monitor disease progression, which is the gold standard for primary efficacy outcomes in clinical trials (Van Eijk et al., 2021).

ALS is predominantly considered as a motor dysfunction disease, and cognitive and behavioral changes are also common in ALS. Up to 50% of patients develop cognitive and/or behavioral impairments such as frontotemporal dementia (FTD) during the disease. Besides, these two diseases share some common genetic mutations and there is a pathological link between familial and sporadic ALS and FTD (Kirola et al., 2022). Some patients with ALS are associated with other neurological diseases, such as Charcot–Marie-Tooth type 2 hereditary neuropathy, Spinocerebellar Ataxia 2, Hereditary Spastic Paraplegia, and so on. This revealed the phenotypic spectrum of neurodegeneration, leading to a better understanding of the genotype–phenotype relationship.

Currently, there are limited treatments for ALS. Riluzole, Edaravone, and AMX0035 are drugs approved by FDA. However, Riluzole can only prolong survival for 2–3 months and Edaravone mildly improves patient mobility, but the effect on survival is unknown (Chia et al., 2018). Thankfully, with a deeper understanding of genetic architecture and disease mechanisms, rapid advances have been made in gene therapies. Now there are clinical trials of antisense oligonucleotides (ASO) underway for ALS patients with SOD1, C9orf72, ATXN2, and FUS mutations. Tofersen targeting SOD1 was shown to be safe and to lower Cerebrospinal fluid (CSF) SOD1 concentrations in a phase 1/2 trial but did not meet its primary endpoint in a phase 3 trial. Other phase 1–3 trials of ASO targeting C9orf72, ATXN2, and FUS are also ongoing.

## 2. Classical amyotrophic lateral sclerosis genes and gene therapies

Although more than 40 genes have been associated with the disease. Mutations in four genes C9ORF72, TARDBP, SOD1, and FUS account for over 70% of FALS cases (Amyotrophic Lateral Sclerosis, 2017), indicating the importance of these genes. Here are the latest developments in the study of the function of these four genes and gene therapies targeting C9ORF72, SOD1, FUS, TARDBP, and ATXN2.

### 2.1. Superoxide dismutase 1

Superoxide Dismutase 1 (SOD1) encodes a cytosolic, Cu/Zn-binding superoxide dismutase and was the first ALS gene to be identified (Rosen et al., 1993). Now more than 200 mutations have been identified, and different mutations were associated with different ages of onset and survival time. For example, the presence of either of the two mutations, G37R and L38V, predicted an earlier age at onset. Mutation A4V (Ala-Val) is correlated with shorter survival (Cudkowicz et al., 1997).

In recent years, gene therapy targeting this gene has some progress in animal experiments. For example, clustered regularly interspaced short palindromic repeat (CRISPR)/CRISPR-associated (Cas9)/sgRNA delivered by the adeno-associated virus (AAV) system can be used for genome editing. Intravenous injection of AAV9-SACas9-SgrNA to neonatal mice with SOD1-G93A mutation can delete the SOD1 gene. The survival rate of mice was improved by 54.6%, and the physiological function was improved as well (Duan et al., 2020). Another group designed a trans-splicing system to introduce nonsense coding substitutions in the mutated SOD1 gene. The authors used dual AAV9 vectors to deliver this system to SOD1-G93A mice and found a 40% reduction in SOD1 inclusions, along with an 11% increase in survival (Lim et al., 2020).

In clinical trials, a recent study found AAV encoding a microRNA (miRNA) targeting SOD1 can suppress the expression of the mutated gene in FALS patients with SOD1 mutation (Mueller et al., 2020). Researchers treated two patients with a single intrathecal infusion of AAV encoding a miRNA targeting SOD1. One of them had transient improvement in the strength of his right leg and lowered SOD1 level in CSF, the other patient had stable scores on a composite measure of ALS function in 12 months. This study indicates that intrathecal miRNA can be used as a potential treatment for SOD1-mediated ALS. Tofersen (BIIB067), an ASO that mediates the degradation of SOD1 messenger RNA (mRNA) to reduce SOD1 protein synthesis, completed phase 3 clinical trials in 2021. The ALSFRS-R scores of ALS patients were not improved in these trials, but the drug could reduce neurofilament and SOD1 protein production (Miller et al., 2020; Mullard, 2021). Another ongoing phase 3 study evaluates the effect of Tofersen when initiated early in 150 presymptomatic carriers of SOD1 mutation with elevated neurofilament (Benatar et al., 2022). On March 22, 2023 Tofersen received a 9–0 vote from the FDA’s advisory committee on Peripheral and central nervous system drugs, and is expected to receive accelerated approval to treat SOD1-ALS patients.

### 2.2. Transactive response DNA-binding protein 43

Transactive response (TAR) DNA-binding protein 43 (TDP-43), encoded by the TARDBP gene, is a DNA/RNA-binding protein. TDP-43 regulates various steps of RNA metabolism, including mRNA splicing, RNA transportation, translation, and miRNA biogenesis (Neumann et al., 2006; Ling et al., 2015; Zuo et al., 2021). In about 97% of ALS patients’ cells, TDP-43 is depleted from the nucleus and found as hyperphosphorylated, aggregated cytoplasmic inclusions (Prasad et al., 2019). TDP-43 pathology is also found in 50% of FTD patients and is seen in nearly all ALS-FTD spectrum cases. There is a link between UNC13A genetic variants (risk factors for FTD and ALS) and loss of TDP-43 function (Ma et al., 2022). One study shows that patients with ALS exhibit mild cognitive deficits in executive functions, language, and fluency, without dementia, all of whom had TDP-43 pathology in extra-motor brain regions (Gregory et al., 2020).

The aggregation sequesters the normal function of TDP-43 and blocks normal cellular processes, finally leading to cell death in a short time. Studies suggest that TDP-43 affects gene expression mainly through the following two pathways: (Brown and Al-Chalabi, 2017) TDP-43 is related to miRNA biosynthesis, so its mutation may influence the production and function of miRNA; (Ryan et al., 2019) aggregation with other RNA-binding proteins affects the expression of other genes. Many of those affected gene products are mitochondrial proteins, and their dysregulation causes a broad mitochondrial imbalance that augments oxidative stress (Suk and Rousseaux, 2020).

As TDP-43 aggregation is a feature of almost all patients with ALS, except those with a mutation in FUS or SOD1. Its aggregates encompass a larger population than those that have the TARDBP mutation. It might be the reason why researchers have not developed treatments that target this gene. Currently, there are trials targeting TDP-43 protein but no trials with ASOs targeting TARDBP. Pozzi et al. (2019) generated single-chain (scFv) antibodies against TDP-43. By virus-mediated delivery into the nervous system, it can mitigate motor defects and TDP-43 proteinopathy in mice expressing ALS-linked TDP-43 mutations. It suggests that antibodies might be new therapeutic avenues for the treatment of ALS and FTD.

### 2.3. Fused in sarcoma

FUS encodes a multifunctional RNA binding protein (RBP), which localizes predominantly to the nucleus under physiological conditions. It is usually involved in transcription, alternative splicing, mRNA transport, mRNA stability, and miRNA biogenesis (Kim et al., 2020). This protein is also located at the neuromuscular junction and is associated with the transcriptional regulation of acetylcholine receptors in the neuromuscular junction (Picchiarelli et al., 2019). In 2009, two groups independently reported that FUS/TLS mutations were associated with FALS (Sreedharan et al., 2008; Vance et al., 2009), and its pathology was similar to that of the gene TARDBP.

A recent study shows that mutant FUS influences functions of other RBPs such as promoting the phase separation of fragile X mental retardation protein (FMRP), another RBP associated with neurodegeneration. This inhibits protein translation (Birsa et al., 2021). FUS toxicity in motor neurons and myotubes will lead to endplate maturation defects, and FUS is directly implicated in neuromuscular junction maintenance and stability (Picchiarelli et al., 2019). Researchers have targeted post-translational acetylation to treat FUS-ALS. Using histone deacetylase (HDAC) inhibitors in the FUS-ALS models can influence cytoplasmic localization of FUS, promote the acetylation of FUS RNA binding domain (RRM) and change its interaction with RNA. FUS acetylation through HDAC inhibitors is presented as a potential therapeutic strategy for FUS-ALS (Tejido et al., 2021). Among teenagers, FUS mutation is associated with more than half of juvenile ALS (JALS) and especially sporadic JALS, providing a new perspective for the diagnosis and new treatment (Chen, 2021).

Jacifusen (ION363), an ASO targeting FUS mutation, was designed and FDA-approved for the experiment in 2019. It consists of a small molecule that is designed to target the FUS mRNA, preventing the production of the FUS protein (Arnold, 2019). It can also reduce the FUS protein considerably in the brainstem tissue (Korobeynikov et al., 2022). Jacifusen has been used to treat another three FUS-ALS patients through the FDA’s compassionate use protocol. A phase 3 trail is ongoing to determine the safety and efficacy of ION363 on 77 patients in American and European cohorts with FUS mutation (Amyotropic Lateral Sclerosis, 2021).

### 2.4. Chromosome 9 open Reading frame 72

Among European and American people, the most common genetic cause of ALS is mutation in the open reading frame 72 (C9ORF72) gene on chromosome 9, accounting for approximately 30% of the cases, 60% in FALS， and 40% in SALS (Van Blitterswijk et al., 2012). The mutation is GGGGCC hexanucleotide repeat expansion in the noncoding region of C9ORF72. The extended repeat is transcribed into RNA, which is then converted into an aberrant protein. Three main disease mechanisms have been proposed: (Brown and Al-Chalabi, 2017) loss of function of the C9ORF72 protein, (Ryan et al., 2019) toxic gain of function from C9ORF72 repeat RNA; (Feldman et al., 2022) dipeptide repeat (DPR) proteins produced by non-ATG translation (DeJesus-Hernandez et al., 2011; Balendra and Isaacs, 2018). Several recent studies point to altered protein homeostasis as one of the fundamental causes of disease pathogenesis. Dysfunction of the autophagy-lysosome pathway synergizes with altered protein homeostasis as one of the fundamental causes of disease pathogenesis (Beckers et al., 2021).

In 2022, an ASO treatment targeting the C9ORF72 mutation came to fruition. Using ASOs in C9-ALS\FTD-derived cells and C9ORF72 BAC transgenic mice, researchers found that they selectively repressed the expression of GGGGCC repeat transcripts. In a patient with mutant C9ORF72 and GGGGCC repeat expansion, CSF polyphosphoric acid levels were significantly reduced after intrathecal ASO. Additional clinical trials will be required (Tran et al., 2022). WVE-004 is an ASO of C9ORF72 mutation, mediating degradation of C9ORF72 mRNAs containing GGGGCC repeat. An ongoing phase 1/2 clinical trial showed that WVE-004 can reduce the poly-GP DPRs in CSF (Wave Life Sciences, 2021). BIIB078 is an antisense of C9ORF72 gene mRNA. However in the phase 1 trial, there was no difference in ALSFRS-R score, slow vital capacity, hand-held dynamometry, or the Iowa oral pressure Instrument between the BIIB078 group and placebo group, so the development of the drug had been discontinued (Study to Assess the Safety, 2020; Hoffman, 2022).

### 2.5. Ataxin 2

Ataxin-2, a polyglutamine (poly Q) protein, is a potent modifier of TDP-43 toxicity in animal and cellular models. In 2010, ATXN2 was found to be associated with up to 4.7% of all ALS (Elden et al., 2010). Ataxin-2 has diverse functions in cells, including RNA processing and receptor endocytosis. What is relevant to ALS are the formation of stress granules (Nonhoff et al., 2007) and induction of aberrant TDP-43 cleavage by caspase (Hart and Gitler, 2012). In a subset of 1,362 patients with ALS, Glass et al. (2022) confirmed that ≥31 polyQ repeats in ATXN2 increased the risk for ALS, and the risk for ALS with FTD was even greater. Another group identified a 9-base pair duplication in the 2-gene ATXN2 sense/antisense region, which can decrease the age at onset for both spinocerebellar ataxia 3 (SCA3) and C9ORF72-ALS (Laffita-Mesa et al., 2021).

ATXN2 is not only associated with ALS but also a powerful modulator of neurological diseases (Laffita-Mesa et al., 2021). For instance, motor neuron disease spinocerebellar ataxia 2 (SCA2) is caused by over 32 CAG repeats in ATXN2. SCA2 is an autosomal dominant lethal disease, which mainly affects the cerebellum, pons, olive, brainstem, frontal lobe, medulla oblongata, cranial, and peripheral nerves. The main clinical manifestations of patients include progressive gait ataxia, dysarthria, dysphagia, hyporeflexia, cognitive decline, saccade slowing, ophthalmoplegia, Parkinsonism, pyramidal features, and/or neuropathy (Lastres-Becker et al., 2008). Peripheral nerve disease such as familial amyloidosis polyneuropathy is also associated with ATXN2.

In the ALS mouse model, researchers administered ASOs targeting ataxin-2 to the central nervous system of TDP-43 transgenic mice and found it could extend the survival rate (Becker et al., 2017). It might benefit patients with ATXN2 mutation because TDP-43 aggregation is a component of nearly all cases of ALS and TDP-43 localization to ATXN2-dependent stress granules is a shared pathologic endpoint. BIIB105 is an ataxin-2 antisense that can degrade ataxin-2 mRNA and reduce the level of ataxin-2 protein. A phase 1 clinical trial is ongoing to evaluate the safety and tolerability of BIIB105 (Biogen, 2020).

Here we summarize the gene therapies targeting C9ORF72, SOD1, FUS and ATXN2 in Table 2.

**Table 2:** Gene therapies targeting C9orf72, SOD1, FUS and ATXN2.

Target Gene	Drug	Company	properties	Aim	Result	Phase	References
SOD1	Tofersen	Biogen; Ionis	ASO	Evaluate the efficacy, safety, tolerability, pharmacokinetics and pharmacodynamics	Ongoing	3	ClinicalTrials.gov: NCT02623699; (Nicolas et al., 2018)
FUS	Jacifusen	Ionis	ASO	Evaluate the efficacy, safety, pharmacokinetics and pharmacodynamics	Ongoing	3	ClinicalTrials.gov: NCT04768972; (Ling et al., 2015)
C9ORF72	BIIB078	Biogen	ASO	Evaluate the safety, tolerability, and pharmacokinetics	Safe, no benefit	1	ClinicalTrials.gov: NCT03626012
	WVE-004	Wave Life Sciences Ltd.	ASO	Evaluate the safety, tolerability, pharmacokinetics and pharmacodynamics	Ongoing	1b/2a	ClinicalTrials.gov: NCT04931862
ATXN2	BIIB105	Biogen	ASO	Evaluate the safety, tolerability, and effect on disease progression	Ongoing	1	ClinicalTrials.gov: NCT04494256

ASO, Antisense Oligonucleotide.

## 3. New causative genes of amyotrophic lateral sclerosis reported since 2016

**Figure 1:**
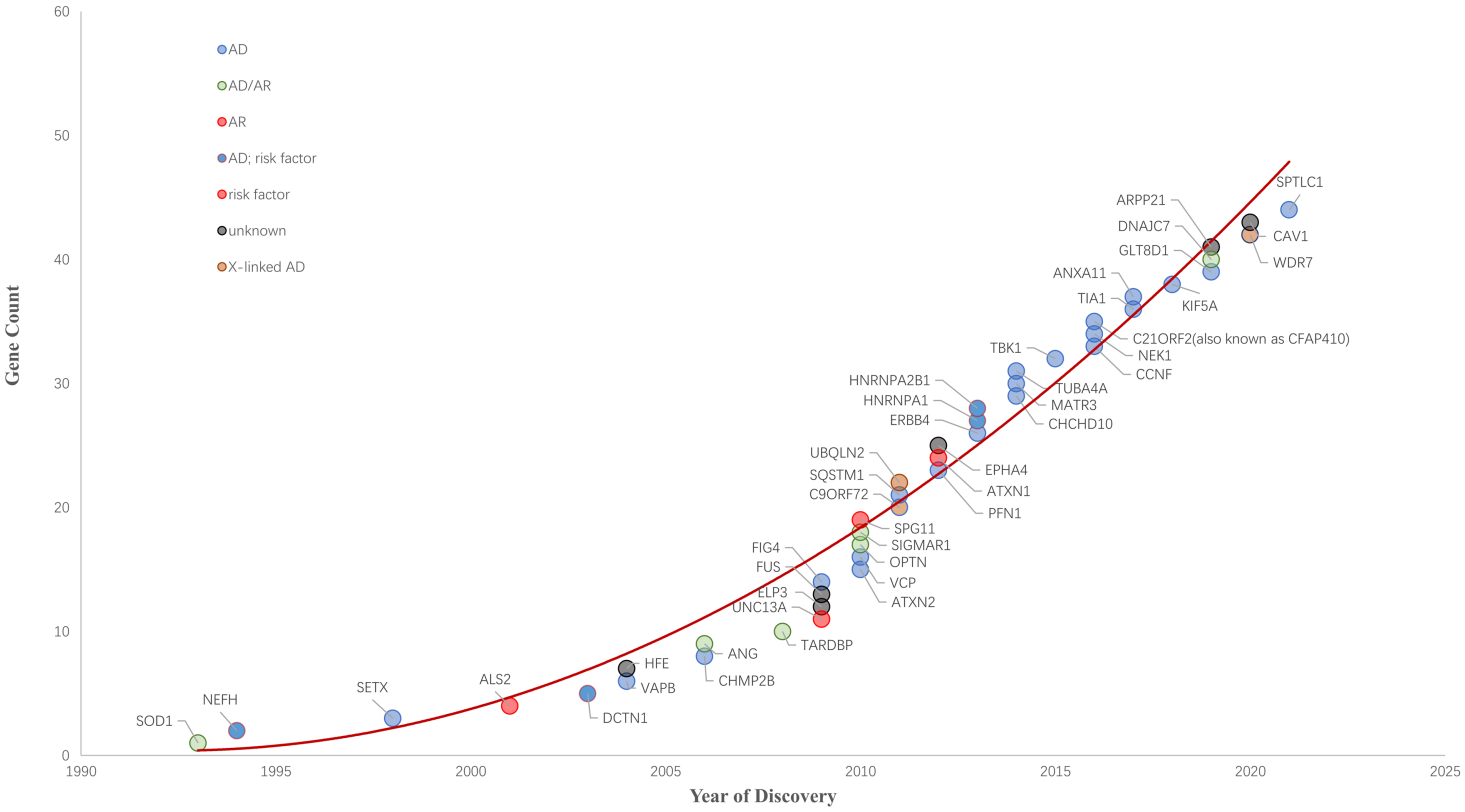
ALS Gene Discovery from 1990 to 2022. The cumulative number of ALS-related genes discovered is growing rapidly. ALS-related genes are plotted and their respective inheritance patterns are represented by different colored circles.

Since 1993 variants in more than 40 genes (SOD1, NEFH, SETX, ALS2, DCTN1, HFE, VAPB, ANG, CHMP2B, TARDBP, UNC13A, ELP3, FUS, ATXN2, OPTN, SPG11, VCP, C9ORF72, SQSTM1, UBQLN2, ATXN1, EPHA4, PFN1, HNRNPA1,CHCHD10, MATR3, TUBA4A, TBK1, CCNF, NEK1, C21ORF2, TIA1, ANXA11, KIF5A, GLT8D1, ARPP21, DNAJC7, WDR7, CAV1, SPTLC1 and so on) have been shown to cause ALS, increase ALS risk or be linked to a difference in the clinical phenotype of Amyotrophic Lateral Sclerosis (n.d.). ALS Gene Discovery from 1990 to 2022 was summaried as Figure 1. Now, We focus on the new genes identified by WES or GWGS and other new techniques after 2016.

### 3.1. NIMA-related kinase 1 and chromosome 21 open reading frame 2

Through WES of ALS patients, NEK1 was identified as an ALS gene in 2016 by Kenna et al. (2016). In the same year, Brenner’s et al. (2016) team also identified NEK1 as a hit through a study of 2000 ALS patients. NEK1 is a widely expressed multi-functional kinase linked to multiple cellular processes. It is involved in the control of cell cycles, DNA damage repair, and cilia formation. There is evidence that NEK1 has an association with FALS and SALS. NEK1 risk variants were identified in nearly 3% of ALS cases, among which 1% are confirmed heterozygous loss of function (LOF) variants (Brenner et al., 2016; Kenna et al., 2016).

It is known that NEK1 interacts with two proteins previously found to be widely expressed and associated with ALS -- the RAB guanine nucleotide exchange factor ALS2 and the endoplasmic reticulum protein VAPB involved in lipid trafficking to the plasma membrane (Cirulli et al., 2015). DNA damage is a feature of NEK1-ALS, for example, there is increased γH2AX in NEK1-ALS iPSC-derived motor neurons (Kok et al., 2021) and NEK1 knockdown has also been shown to lead to increased morphological signs of DNA damage (Chen et al., 2011).

Researchers have used direct nucleotide sequencing in 377 ALS patients to test the frequency of NEK1 mutations in a Chinese population. The results show 2.7% of all ALS patients carried NEK1 risk variants. The frequency of novel heterozygous LOF mutation presented as 0.8%. All in all, the frequency of NEK1 LOF mutations is low in mainland China (Shu et al., 2018). A study on the clinical manifestations of patients with LOF variants of NEK1 ALS was also conducted in Taiwan, China. The result showed patients with a NEK1 LOF variant tend to have a higher frequency of hand-onset disease, compared with ALS patients without a NEK1 LOF variant (Tsai et al., 2020). Recently one study found NEK1 deficiency disrupts proteome homeostasis in the CNS, which can be ameoriated by inhibition of RIPK1. They suggested the possibility of inhibiting RIPK1 kinase for the treatment of ALS (Wang et al., 2021).

Through whole-genome-sequencing in a great number of (12,577 cases and 23,475 controls, combined with 2,579 cases and 2,767 controls in an independent replication cohort) ALS patients, Van Rheenen et al. (2016) identified C21ORF2 (also known as CFAP410) as a gene associated with ALS risk. The C21ORF2 protein is found to interact with NEK1. It is part of the cilia, involved in DNA damage response and repair, and mitochondrial function (Fang et al., 2015; Watanabe et al., 2020). Recent studies found that C21ORF2 V58L mutation in motor neurons induced from mouse embryonic stem cells impaired neurite outgrowth, suggesting the inhibition of NEK1 activity to be a potential therapeutic approach for ALS associated with C21ORF2 mutation (Watanabe et al., 2020).

### 3.2. Cyclin F

In 2016, Kim et al. (2016) identified CCNF as the causative gene for ALS by WES analysis of a large Australian family with ALS, frontotemporal dementia, or both diseases. Its inheritance follows an autosomal dominant pattern. Patients usually present with either ALS with FTD or FTD alone. CCNF encodes cyclin F, a component of an E3 ubiquitin-protein ligase complex. This complex is responsible for tagging proteins with ubiquitin and marking them for degradation *via* the ubiquitin-proteasome system (D’Angiolella et al., 2010), and mutations in CCNF are associated with hyper-ubiquitylation of proteins and defects in ubiquitination processing (Lee et al., 2018). This finding suggests that mutations in CCNF may lead to abnormal protein inhibition, so treatments that enhance protein clearance or reduce ubiquitination may be viable treatments (Chia et al., 2018). CCNF is closely associated with RNA-binding proteins linked to ALS/FTD, including splicing factor proline and glutamine-rich (SFPQ), which is a pathological hallmark of ALS/FTD (Rayner et al., 2021). And neuronal cells overexpressing mutant CCNF show an increase in ubiquitin-tagged proteins, including TDP43 (Kim et al., 2016). CCNF also affects axon growth which is identified in zebrafish models (Hogan et al., 2017). Loss of function of cyclin F has recently been found to affect heat shock proteins 90 (HSP90), which is a possible Cyclin-F LOF mediated chaperone dysregulation that might be relevant to ALS (Siebert et al., 2022). Among Australian, American, European, and Asian populations with FALS-FTD, the CCNF mutation frequency ranged from 0.6 to 3.3% (Kim et al., 2016). The mutation rate in Chinese SALS is low, about 0.8% (Wei et al., 2019), and the frequency of mutations in Taiwan is also approximately 0.8% (Tsai et al., 2018).

### 3.3. Annexin A11

In 2017, Smith et al. (2017) sequenced 751 patients’ whole exons with familial ALS, and results showed that the vesicle transporter annexin A11 (ANXA11) was associated with ALS. ANXA11 mutations in FALS and SALS cluster in the N terminus, which implicates a functional impact of mutations in this region. ANXA11 encodes the protein AnnexinA11, which is a calcium-dependent phospholipid-binding protein. Mutant ANXA11 forms insoluble protein aggregates in neurons and produces neurotoxicity. Mutant protein does not bind calcyclin, and disruption of this binding is disease-specific. Recent studies have found that mutations in this gene also damage intracellular Ca^2+^ homeostasis and influence stress granule dynamics (Nahm et al., 2020), impairing RNA granule transport by disrupting their interactions with lysosome (Liao et al., 2019). ANXA11 mutations lead to intracellular Ca^2+^ homeostasis dysregulation and abnormal protein aggregation, which may lead to MN death, and based on these Nahm et al. (2020) suggest that a multi-target therapeutic strategy may be an ideal integrative approach for more effective ALS management. Studies have linked the gene not only to ALS but also to other diseases such as FTD. So this gene should be considered the cause of a novel multisystem proteinopathy (Leoni et al., 2021). In China, the mutation frequency of this gene was 5.6% (1/18) in FALS patients, 2.3% (8/353) in SALS patients, and 8.3% (1/12) in ALS-FTD patients, indicating that the ANXA11 gene is one of the most frequently mutated genes in Chinese patients with ALS (Zhang et al., 2018). Clinically, ALS cases carrying the p.D40G mutation have shown late onset and the presence of cytoplasmic immunoreactive inclusions in postmortem tissue (Smith et al., 2017).

### 3.4. Cytotoxic granule-associated RNA binding protein

Mackenzie et al. (2017) using WES in a European family with ALS-FTD, first discovered Cytotoxic Granule-Associated RNA-Binding Protein (TIA1) as an ALS-related gene. This gene encodes an RNA-binding protein with a C-terminal low-complexity sequence domain, which is similar to TDP-43 or FUS (Mackenzie et al., 2017). Postmortem neuropathology in TIA1 mutation carriers revealed numerous rounds, clear, TAR DNA-binding protein 43-positive inclusions with consistent pathological features. TIA1 mutation affects stress particle dynamics and leads to TDP-43 accumulation. TDP-43 recruited to these stress granules becomes immobile and insoluble (Mackenzie et al., 2017; Purice and Taylor, 2018). However, research has cast doubt on TIA1’s role in ALS. In a large patient-controlled series of studies in Europe, researchers sequenced the coding region of TIA1 in 693 FTD, 341 ALS, and 86 ALS-FTD patients as well as 1,039 controls. Five rare heterozygous missense variants were observed, but only one of these variants was absent from the control cohort (Baradaran-Heravi et al., 2018). It suggested the exact genetic contribution of TIA1 to ALS and FTD pathogenesis remains to be further elucidated.

A clinical study targeting mutations in the TIA1 gene found that TIA1 mutation carriers developed ALS with or without FTD and without other neurological or psychiatric features. In pathological HE staining, the number of lower motor neurons containing round eosinophils and Lewy body-like inclusions was increased (Hirsch-Reinshagen et al., 2017), which might be a distinctive feature of ALS caused by TIA1 mutations. In Chinese patients with SALS, the mutation frequency of TIA1 was 0.14%, which suggests that TIA1 mutation is an uncommon genetic cause for ALS in the Chinese population (Gu et al., 2018).

### 3.5. Kinesin family member 5A

Through GWAS and rare variant burden analysis, kinesin family member 5A (KIF5A) was identified as a novel gene associated with ALS in 2018 by Nicolas et al. (2018). KIF5A belongs to a family of motor proteins, kinesins, which are selectively expressed in neurons and have been implicated in the transport of intracellular organelles, such as particles in axons and dendrites that interact with RNA and RNA-related molecules. Mutations in KIF5A can cause a variety of diseases. For instance, mutations in the N-terminal domain can lead to hereditary spastic paraplegia (SPG10) or Charcot–Marie-Tooth type 2 hereditary neuropathy (CTM2) (Reid et al., 2002; Liu et al., 2014), and the lack of KIF5A expression can lead to accumulations of downstream proteins, which are associated with neurodegeneration in patients with multiple sclerosis (MS) (Hares et al., 2021). ALS-associated mutations are located at the C-terminal domain (Nicolas et al., 2018). KIF5A mutations cause cell localization errors, altered axonal transport, and disruption of neuronal homeostasis (Baron et al., 2022). Oligomers have been found to form easily in mouse models, and this toxic acquired mutation can also induce neuronal toxicity (Nakano et al., 2022), which was also found in patients with IPSC-derived motor neurons (Pant et al., 2022). Analysis of KIF5A sequences in a large ALS cohort in China showed that the frequency of KIF5A mutations accounted for 0.16% of SALS patients in China (Zhang et al., 2019). Patients with clinical LOF mutations show prolonged survival relative to typical ALS cases (Nicolas et al., 2018).

### 3.6. DNAJ heat shock protein family member C7

Farhan et al. (2019) identified DNAJC7 as an ALS-associated protein by WES. It encodes a member of the heat-shock protein family, HSP40. Along with HSP70 proteins, they regulate the folding, misfolding, and clearance of other proteins and peptides to promote protein homeostasis. A higher incidence of the significant protein truncation variant PTV (present in SOD1, FUS, and NEK1) was observed in DNAJC7 mutant cases. The pathogenic mechanism of this gene mutation may be inhibition of proper HSP90AB1 function resulting in the accumulation of TDP-43, destabilization of interactions with other J-proteins involved in neuroprotection, or the inability to accurately chaperone the variety of Hsp70s or Hsp90s involved in ALS-associated protein aggregate (Dilliott et al., 2022). Clinically, mutations of DNAJC7 are rare in Chinese ALS patients (Sun et al., 2021).

### 3.7. Glycosyltransferase 8 domain containing 1

In 2019, Cooper-Knock et al. (2019) reported GLT8D1 mutation is associated with ALS. GLT8D1 encodes a widely expressed glycosyltransferase of unknown function that may be involved in the synthesis of signaling molecules, such as gangliosides. Mutations can reduce enzyme activity and this mechanism of loss of function is an attractive therapeutic target (Moll et al., 2020). The p.R92C mutation was co-segregated with the disease, and both R92C and G78W changes impair GLT8D1 enzyme activity. Mutated GLT8D1 in the zebrafish model exhibited *in vitro* cytotoxicity, further indicating the pathogenicity of the GLT8D1 mutation (Cooper-Knock et al., 2019). A Chinese study has not found an association between GLT8D1 and ALS yet (Yilihamu et al., 2021), so has Australia (Chan Moi Fat et al., 2021). Based on the research into the downstream mechanisms underlying neurotoxicity caused by mutations in this gene, Moll et al. (2022) suggest that mutations in this gene disrupt ganglioside homeostasis and cause disrupted neurotrophin signaling. It pathological pathway is similar to CAV1, suggesting a potential new therapeutic approach *via* upregulation of GLT8D1.

### 3.8. cAMP regulated phosphoprotein 21

In an autosomal-dominant ALS pedigree, Cooper-Knock et al. (2019) found that GLT8D1 p.R92C mutation and ARPP21 p.P529L may have a synergistic effect for ALS in a cohort of European descent. Patients in the context of both mutations are more severe than in the presence of either mutation in isolation. However, this observation has only been validated in the other two ALS cohorts and did not find a significant association between ALS and ARPP21. In an Australian cohort using whole-exome and whole-genome sequencing data, no novel mutations were identified in ARPP21 (Chan Moi Fat et al., 2021). Another Chinese study found 25 rare variants of ARPP21 in the patients and controls but did not find a significant association between ALS and ARPP21 (Li et al., 2020). These suggest that GLT8D1 and ARPP21 mutations are not a common cause of ALS in Australian and Chinese familial and sporadic cohorts.

### 3.9. Caveolin 1

Using rare variant burden analysis within enhancers, Cooper-Knock et al. (2021) identified CAV1 as an ALS risk gene in 2020. The CAV1 protein is encoded by the CACNA1C gene. In motor neurons CAV1 express in a hetero-oligomeric complex within membrane lipid rafts (MLRs) on the cell surface and has a key role in the organization of intercellular signaling. In ALS patients, CAV1 enhancers and coding sequences aggregate, resulting in decreased CAV1 expression and disruption of MLRs. This pathogenic pathway is similar to GLT8D1 and both of them result in disrupition in MLRs, suggesting up-regulation of CAV1 to restabilish of MLRs has therapeutic potential (Sawada et al., 2019). Abnormal activity of CAV1 channels can disrupt intracellular calcium homeostasis (Li et al., 2021). Generally, identification of the ALS gene is focused on the coding region, however, disease-associated mutations in this gene are located within enhancer and coding regions. This identification method will help to find more genetic factors.

### 3.10. WD repeat domain

Tandem repeats have a relationship with human-specific traits. Course et al. screened human-specific intronic variable number tandem repeats (VNTRs) and found an enrichment of a higher copy number of a specific variable number tandem repeats in three independent cohorts of ALS patients (Course et al., 2020). It is located at the last intron of WDR7. VNTRs on WDR7 are long, amplified in large numbers, and varying. Its amplification direction ranges from the 3′ end to the 5′ end. Complementary sequences form stable hairpins, possibly miRNAs and aggregate in the cytoplasm. High VNTR copy number leads to neurodegenerative diseases by affecting the function of synaptic transmission. The amplification of these repeats can be traced back to the ancient human genome. WDR7 may interact with other ALS-causing genes such as FUS and TDP-43.

### 3.11. Serine palmitoyl transferase long-chain base subunit 1

In 2021, two research teams reported SPTLC1 as a new pathogenic gene for ALS (Johnson et al., 2021; Mohassel et al., 2021). Mohassel et al. (2021) sequenced the genomes of 11 ALS patients in 7 independent families, and identified four SPTLC1 variants in seven JALS. Johnson et al. (2021) used WES in a total of 66 patients with juvenile ALS and 6,258 adult patients with ALS. They found 3 unrelated JALS patients carried *de novo* variants in SPTLC1 and identified a JALS patient with another SPTLC1 variant for whom inheritance could not be determined. The pathogenic mechanism of this gene may be the disruption of normal homeostasis of serine palmitoyl transferase (SPT). The study showed that p.Ala20Ser variation can lead to increased aberrant utilization of alanine and glycine as substrates, causing the disease in altering the SPT amino acid substrate. Interestingly, almost all cases associated with mutations in this gene are juvenile, which may suggest that this gene is an important causative factor in JALS.

## 4. Discussion and conclusion

**Figure 2:**
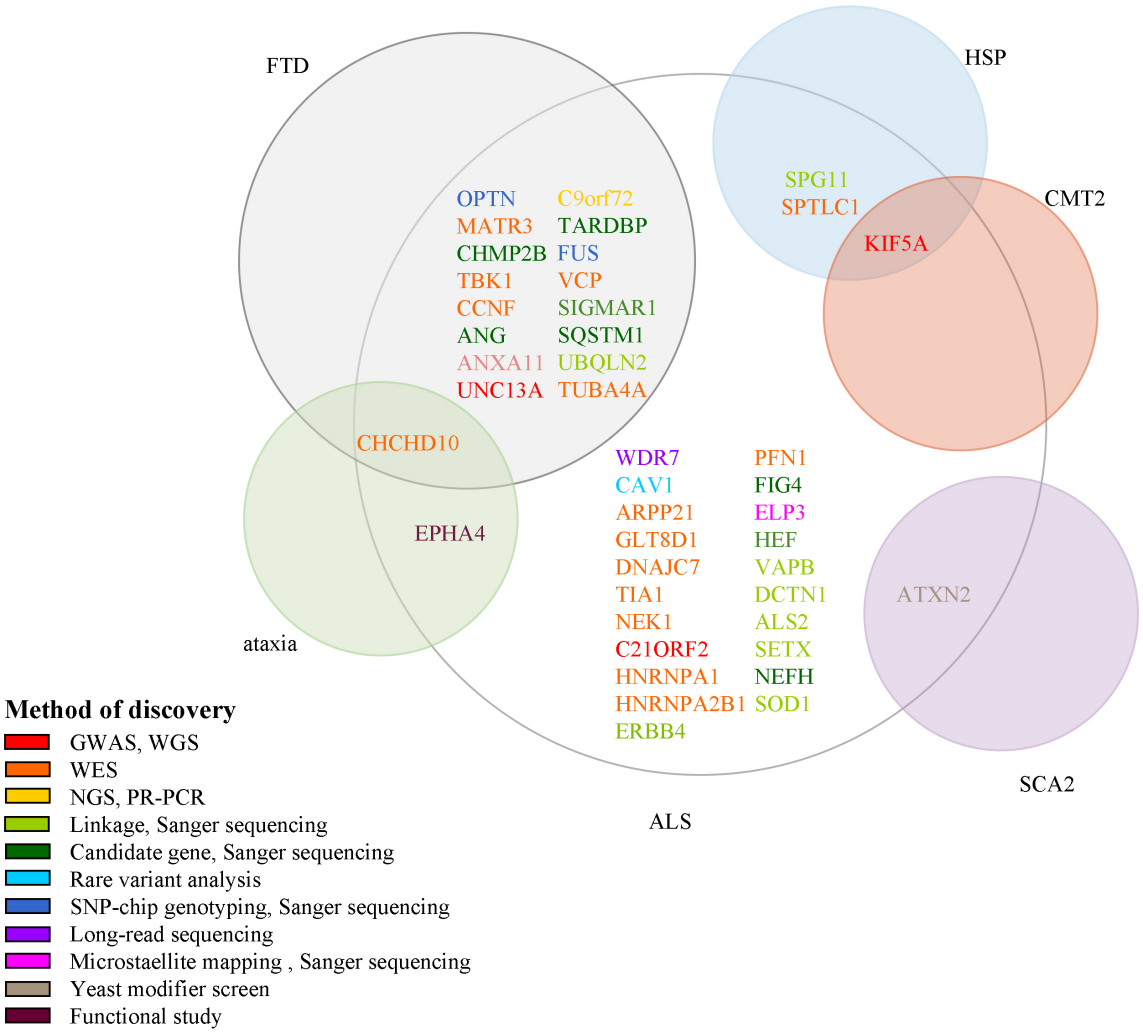
Methods for the discovery of ALS pathogenic genes and their association with other neurological diseases. Each circle indicates one disease and its name is presented beside it in red color. There are 15 genes associated with both ALS and FTD; 1 gene associated with ALS, ataxia, and FTD; 1 gene associated with both ALS and SCA2; 1 gene associated with both ALS and HSP; 1 gene associated with ALS, HSP, and CMT2. ALS, amyotrophic lateral sclerosis; GWAS, genome-wide association study; WGS, whole-genome sequencing; WES, whole-exome sequencing; NGS, next-generation sequencing; RP-PCR, repeat-primed polymerase chain reaction; SNP, single nucleotide polymorphism.

In this review, we summarize 5 classic and 12 new genes associated with ALS, and plot ALS-associated genes according to their discovery method and overlay disease in Figure 2. ALS is one of the few neurodegenerative diseases approved for disease-modifying therapy. At present, there have been only a few clinical trials of gene therapies targeting ALS genes, including SOD1, FUS, C9ORF72, and ATXN2. However, with the continuous discovery of new pathogenic genes, the pathogenic mechanism is gradually clear, and new possible treatment strategies are constantly proposed, which provides hope for the treatment of ALS patients. Among the classic genes, two gene therapy drugs targeting SOD1 and FUS (tofersen and jacifusen) have entered phase 3 clinical trials. Although tofersen missed the primary endpoint in a first phase 3 trial, it still lowered the levels of SOD1 protein and an experimental biomarker of neuroinflammation (Miller et al., 2020; Mullard, 2021). It makes us rethink if the patients were treated early enough and the duration of trials was long enough. Besides, in clinical trials, tofersen appears to be safe in the treated patients, so it might be important for ASOs. As ALS is a genetically heterogeneous complex disease, treatments targeting specific mutations might provide more benefits to patients than general treatments. Therefore, the development of new gene therapies and the identification of novel ALS-related genes will be vital to the treatment and prevention of ALS. New genetic discovery mainly depended on technological developments such as GWAS and WES. Since 2016, 12 novel genes–ANXA11, ARPP21, CAV1, C21ORF2, CCNF, DNAJC7, GLT8D1, KIF5A, NEK1, SPTLC1, TIA1, and WDR7 have been identified using these techniques, which laid the ground for personalized, gene-specific therapeutic approaches. More recent method advancements have driven research progress, for example, a machine learning model RefMap integrates epigenetic data with GWAS summary statistics for gene discovery. Besides, their data and method are unique with respect to the depth and number of assessments and they provided a general framework that can be applied to the identification of risk genes involved in a wide range of complex diseases and traits (Zhang et al., 2022). Perhaps the new method will help us to further understand the pathogenesis of ALS and provide new ideas for the treatment of the disease.

Some genes are associated with multiple diseases too; for example, CCNF and ANXA11 are linked to FTD; KIF5A is linked to SPG10 and CMT2; ANXA2 is linked to SCA2. So studies of the pathogenic mechanisms of these genes will benefit the understanding of a variety of neurological diseases.

The mutant frequencies of ALS genes are different across different ethnicities. C9ORF72 is the most common genetic cause of ALS among European and American populations, accounting for approximately 30% of the cases (Van Blitterswijk et al., 2012). The mutation frequency of this gene is 46% in FALS and 21.6% in SALS of the Polish population. However, in Asian populations, the mutation frequency of C9ORF72 is 2.3% in FALS and 0.3% in SALS. Unlike C9ORF72, SOD1 mutations are more prevalent in Asian populations than in European populations. These demonstrated that the genetic architecture of ALS is different between Asian populations and European populations. Therefore, it is necessary to investigate the frequency and clinical characteristics of newly identified genes in different ethnic groups, and appropriate consideration is needed when performing genetic testing of patients with ALS.

## Author contributions

HW: literature search, writing-original draft preparation, and writing-reviewing and editing. LG: writing-reviewing and editing. MD: study conception and design, and writing-reviewing and editing. All authors contributed to the article and approved the submitted version.

## Funding

The study was funded by the National Natural Science Foundation of China (No. 82273915) and the Beijing Natural Science Foundation (No. 7192223).

## Conflict of interest

The authors declare that the research was conducted in the absence of any commercial or financial relationships that could be construed as a potential conflict of interest.

## Publisher’s note

All claims expressed in this article are solely those of the authors and do not necessarily represent those of their affiliated organizations, or those of the publisher, the editors and the reviewers. Any product that may be evaluated in this article, or claim that may be made by its manufacturer, is not guaranteed or endorsed by the publisher.
